# Non-Invasive Ventilation Failure in Pediatric ICU: A Machine Learning Driven Prediction

**DOI:** 10.3390/diagnostics14242857

**Published:** 2024-12-19

**Authors:** Maria Vittoria Chiaruttini, Giulia Lorenzoni, Marco Daverio, Luca Marchetto, Francesca Izzo, Giovanna Chidini, Enzo Picconi, Claudio Nettuno, Elisa Zanonato, Raffaella Sagredini, Emanuele Rossetti, Maria Cristina Mondardini, Corrado Cecchetti, Pasquale Vitale, Nicola Alaimo, Denise Colosimo, Francesco Sacco, Giulia Genoni, Daniela Perrotta, Camilla Micalizzi, Silvia Moggia, Giosuè Chisari, Immacolata Rulli, Andrea Wolfler, Angela Amigoni, Dario Gregori

**Affiliations:** 1 Unit of Biostatistics, Epidemiology and Public Health, Department of Cardiac, Thoracic, and Vascular Sciences and Public Health, University of Padova, Via Loredan 18, 35131 Padova, Italy; mariavittoria.chiaruttini@studenti.unipd.it (M.V.C.); giulia.lorenzoni@unipd.it (G.L.); 2 Pediatric Intensive Care Unit, Department of Women’s and Children’s Health, University Hospital of Padova, Via Giustiniani 3, 35128 Padova, Italy; marco.daverio@aopd.veneto.it (M.D.); luca.marchetto@aopd.veneto.it (L.M.); angela.amigoni@aopd.veneto.it (A.A.); 3 Pediatric Intensive Care Unit, Buzzi Children’s Hospital, Via Lodovico Castelvetro 32, 20154 Milan, Italy; francesca.izzo@asst-fbf-sacco.it; 4 Department of Anesthesia Resuscitation Emergency Care, Fondazione IRCCS Ca’ Granda Ospedale Maggiore Policlinico di Milano, Via Francesco Sforza 35, 20122 Milan, Italy; giovanna.chidini@policlinico.mi.it; 5 Pediatric Intensive Care Unit, Pediatric Trauma Center, Fondazione IRCCS Policlinico Universitario “A. Gemelli”, Largo Agostino Gemelli 8, 00136 Rome, Italy; enzo.picconi@yahoo.it; 6 Anaesthesia and Pediatric Resuscitation, AOU Alessandria, SS Antonio e Biagio e Cesare Arrigo Hospital, Spalto Marengo 43, 15121 Alessandria, Italy; cnettuno@ospedale.al.it; 7 Pediatric Intensive Care Unit, San Bortolo Hospital, Viale Ferdinando Rodolfi 37, 36100 Vicenza, Italy; elisa.zanonato@aulss8.veneto.it; 8 Anesthesia and Resuscitation Unit, IRCCS Burlo Garofolo, Via dell’Istria 65, 34137 Trieste, Italy; raffaella.sagredini@burlo.trieste.it; 9 Anaesthesia, Emergency and Pediatric Intensive Care Unit, Bambino Gesu’ Children Hospital IRCCS, Piazza di Sant’Onofrio 4, 00165 Rome, Italy; emanuele.rossetti@opbg.net; 10 IRCCS AOU di Bologna, Via Giuseppe Massarenti 9, 40138 Bologna, Italy; cristina.mondi@libero.it; 11 Department of Emergency Acceptance, Bambino Gesù Children’s Hospital, Piazza di Sant’Onofrio 4, 00165 Rome, Italy; corrado.cecchetti@opbg.net; 12 Pediatric and Neonatal Intensive Care Unit, Children’s Hospital Regina Margherita, Piazza Polonia 94, 10126 Turin, Italy; vitale_oirm@yahoo.it; 13 ARNAS G. di Cristina Hospital, 90127 Palermo, Italy; ng.alaimo@gmail.com; 14 Pediatric Intensive Care Unit, Children’s Hospital Meyer, IRCCS, Viale Gaetano Pieraccini 24, 50139 Florence, Italy; denise.colosimo@meyer.it; 15 Paediatric Intensive Care Unit, Azienda Ospedaliera Universitaria Integrata di Verona, Piazzale Aristide Stefani 1, 37126 Verona, Italy; francesco.sacco@aovr.veneto.it; 16 Neonatal and Pediatric Intensive Care Unit, Maggiore della Carità University Hospital, L.go Bellini, 28100 Novara, Italy; genonigiulia@gmail.com; 17 A.R.C.O. Palidoro, Bambino Gesù Children’s Hospital, Piazza di Sant’Onofrio 4, 00165 Rome, Italy; daniela.perrotta@opbg.net; 18 Pediatric and Neonatal Intensive Care Unit, IRCCS G Gaslini, Via Gerolamo Gaslini 5, 16147 Genoa, Italy; camilla.micalizzi@gmail.com; 19 Pediatric Intensive Care Unit, AORN Santobono-Pausilipon, Via della Croce Rossa 8, 80122 Naples, Italy; s.moggia@santobonopausilipon.it; 20 UOSD Pediatric Resuscitation, ARNAS Garibaldi PO Nesima, Piazza Santa Maria di Gesù 5, 95124 Catania, Italy; giosuechisari@msn.com; 21 UOC Neonatal Pathology and TIN, AOU G MARTINO, Via Consolare Valeria 1, 98124 Messina, Italy; 065518@polime.it; 22 Department of Emergency, Division of Anesthesia IRCCS G Gaslini, Via Gerolamo Gaslini 5, 16147 Genoa, Italy; andreawolfler@gaslini.org

**Keywords:** non-invasive ventilation, NIV, NIV failure, PICU, TIPNet, machine learning, predictive models, random forest

## Abstract

**Background/Objectives**: Non-invasive ventilation (NIV) has emerged as a possible first-step treatment to avoid invasive intubation in pediatric intensive care units (PICUs) due to its advantages in reducing intubation-associated risks. However, the timely identification of NIV failure is crucial to prevent adverse outcomes. This study aims to identify predictors of first-attempt NIV failure in PICU patients by testing various machine learning techniques and comparing their predictive abilities. **Methods**: Data were sourced from the TIPNet registry, which comprised patients admitted to 23 Italian Paediatric Intensive Care Units (PICUs). We selected patients between January 2010 and January 2024 who received non-invasive ventilation (NIV) as their initial approach to respiratory support. The study aimed to develop a predictive model for NIV failure, selecting the best Machine Learning technique, including Generalized Linear Models, Random Forest, Extreme Gradient Boosting, and Neural Networks. Additionally, an ensemble approach was implemented. Model performances were measured using sensitivity, specificity, AUROC, and predictive values. Moreover, the model calibration was evaluated. **Results**: Out of 43,794 records, 1861 admissions met the inclusion criteria, with 678 complete cases and 97 NIV failures. The RF model demonstrated the highest AUROC and sensitivity equal to 0.83 (0.64, 0.94). Base excess, weight, age, systolic blood pressure, and fraction of inspired oxygen were identified as the most predictive features. A check for model calibration ensured the model’s reliability in predicting NIV failure probabilities. **Conclusions**: This study identified highly sensitive models for predicting NIV failure in PICU patients, with RF as a robust option.

## 1. Introduction

Non-invasive ventilation (NIV) has garnered increasing attention in recent years as a possible first-step treatment to avoid invasive mechanical ventilation (IMV), particularly in pediatric intensive care units (PICUs) [1,2,3]. NIV provides ventilatory support through external interfaces, such as masks or nasal prongs, in contrast to IMV, which requires endotracheal intubation or tracheostomy. Prolonged IMV, while often necessary, can lead to significant physical and psychological stress, extended recovery times, and increased healthcare costs [4]. In contrast, NIV offers several advantages by minimizing these risks. It preserves natural airway defenses and reduces the incidence of ventilator-induced lung injuries [3,5], such as ventilator-associated pneumonia (VAP) [6], which is associated with high morbidity and mortality rates. NIV also minimizes upper respiratory airway trauma and reduces the need for sedative drugs, which were shown to negatively impact clinical outcomes [7]. These benefits make NIV an attractive option for managing both acute and chronic respiratory failure in critically ill patients.

However, despite its benefits, NIV can occasionally fail. Identifying patients at high risk of NIV failure could help ensure timely and appropriate interventions [8,9]. In fact, delaying the transition from NIV to IMV in patients who do not respond adequately may worsen their condition, prolong hypoxia, and increase the risk of mortality [10,11,12,13].

In the recent years, diverse studies were conducted to evaluate the risk factors associated to NIV failure [14,15,16,17,18,19,20]. However, given the variety of etiologies underlying respiratory failure and the heterogeneity in clinical indications and responses to NIV [11], a comprehensive risk assessment model could benefit to predict the likelihood of NIV failure: through combined (rather than single) consideration of the effects of risk factors, physicians could make more informed decisions about whether to initiate NIV or opt directly for IMV. With this purpose, few studies were conducted using a multivariable logistic predictive model. Some examples were given by Liengswangwong et al. in 2020, who conducted a retrospective cross-sectional, single-center study to develop a clinical scoring system to stratify patients with acute respiratory failure based on the likelihood of NIV failure [21]; Baker et al. in 2021, who performed a single-center retrospective cohort study of children admitted to a quaternary care PICU, who were treated with positive pressure delivered noninvasively, to predict NIV failure by adjusted logistic and Cox regressions [8]; and Stefan et al. in 2021, who developed and validated a clinical risk prediction score for NIV failure on a retrospective cohort from a multi-hospital electronic health record database, considering non-surgical patients [22].

Nevertheless, statistical methods, such as machine learning techniques (MLTs), capable of handling a large number of data and interacting predictors may be appropriate. In the literature, there are some published papers such as those by Feng et al. in 2021, who proposed a dynamic prediction of late NIV failure in an intensive care unit, using a Time Updated Light Gradient Boosting Machine (TULightGBM) model [23]. Another example was given by Pappy et al. in 2022, who developed a predictive model for high-flow nasal cannula failure in an intensive care unit using a recurrent neural network with transfer learning [24]. Finally, Bose et al. in 2023, built a data-driven model for early prediction of the need for IMV on an observational cohort of 13,651 PICU patients using Extreme Gradient Boosting (XGBoost) and a convolutional neural network (CNN) [25].

However, as most of the proposed predictive models are based on a single-center population, our study aimed to propose a systematic evaluation of standard MLTs on a large multi-center registry of patients, including several variables related to admission and the first hour in PICUs. Furthermore, since NIV failure is relatively rare during PICU stays [26], comparing models in terms of sensitivity rather than accuracy should clarify the performance of MLTs in the context of low event rates [27].

## 2. Materials and Methods

### 2.1. Data Source

PICU admission data were extracted from the Italian Network of Pediatric Intensive Care Units (TIPNet) registry. TIPNet is a research network involving more than 20 Italian PICUs. Study data were collected and managed using REDCap electronic data capture tools hosted at University of Padova [28]. REDCap (Research Electronic Data Capture) is a secure, web-based software platform designed to support data capture for research studies, providing (1) an intuitive interface for validated data capture; (2) audit trails for tracking data manipulation and export procedures; (3) automated export procedures for seamless data downloads to common statistical packages; and (4) procedures for data integration and interoperability with external sources. Data were anonymized at the moment of data extraction.

### 2.2. Study Population

We included patients admitted to 23 Italian PICUs in the period between 1 January 2010 and 1 January 2024. Inclusion criteria considered patients with respiratory failure as their principal diagnosis, aged 31 days to 16 years, who underwent NIV as their first ventilation technique at PICU admission. Patients who started NIV before PICU admission or in the days following admission were excluded as we only had predictive variables from the admission form and the first hour in PICU. Only the first admission of each patient was selected. In addition, we opted for listwise deletion technique to handle missing data, excluding cases with missing values in outcome or predictors—under the assumption of missing data completely at random (MCAR)—as the difference in the characteristics of the population with NIV failure between overall cases and complete cases only was not statistically significant.

### 2.3. Outcome and Predictors

The outcome of interest is the failure of the first NIV attempt during PICU stay defined as the initiation of IMV after the NIV attempt or the transition to a different NIV delivery device due to the presence of at least one of the following clinical conditions: (i) hypercapnia, (ii) hypoxia, (iii) excessive secretions, (iv) ineffective cough, (v) discomfort/agitation, and (vi) intolerance. Demographics (sex, age, ethnicity, weight), medical history (chronic disease, the presence of bronchiolitis/asthma), characteristics at presentation (Paediatric Overall Performance Category (POPC), priority of admission, state of consciousness, systolic blood pressure (SBP), the fraction of inspired oxygen (FiO_2_), the base excess), and methods of NIV administration (nasal mask, oronasal mask, nasal cannulas, helmet, full-face mask) were extracted and included as candidate predictors for the analysis. Prior to inclusion in the models, their correct structures were checked to have binary, categorical and continuous data as appropriate.

### 2.4. Ethic Committee Approval

All investigations were carried out following the Declaration of Helsinki. This study is part of a multiple research program based on TIPNet Registry, approved on 23 May 2014 by the Ethic Committee of Milano Area C—Niguarda Cà Granda Hospital (protocol number 269-052014).

### 2.5. Machine Learning Techniques

We constructed predictive models for NIV failure during the PICU stay using the most common standard ML approaches: (i) the generalized linear model (GLM) [29], the random forest (RF) [30], the XGBoost [31], and neural network models (NNET) [32].

Moreover, the ensemble (SuperLearner) algorithm was used, defined as the optimal weighted average of the previous models. Particularly, the algorithm applies the Nelder–Mead method via the optim function to minimize rank loss [33].

The logistic regression, specified as Generalized Linear Model as follows, is known for its simplicity and interpretability [29], where *g*(.) is the link function applied to the mean of the response variable (π), *X* is the matrix of predictors, and *β* the vector of coefficients:

$$g(\pi )=\log(\frac{\pi }{1-\pi })=X\beta .$$

The logistic regression is straightforward to understand and provides coefficients that can be easily interpreted to understand the relationship between predictors and the outcome. Additionally, logistic regression has a solid theoretical foundation and are widely used in medical research to predict the probability of clinical events. However, it assumes a linear relationship between predictors and the outcome, which may not capture complex patterns in the data unless explicitly modeled.

On the other hand, supervised learning methods such as RF and XGBoost can capture complex interactions and non-linear relationships between predictors and the outcome, even non explicitly modeled, but at the price of lower interpretability [31]. However, they provide measures of variable importance (VIMP) [34], which can help understand the contribution of each predictor.

**Figure 1:**
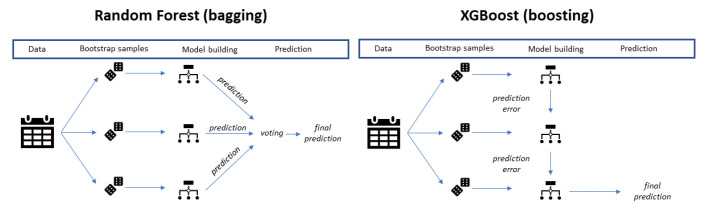
Bagging vs. boosting algorithm.

XGBoost (boosting algorithm) differs from RF (bagging algorithm) since it uses a gradient boosting framework that builds trees sequentially in such a way as to ensure that the errors of the previous tree are reduced; on the contrary, RF builds trees parallelly and combines results at the end of the process training of each classifier (Figure 1) [30]. Moreover, the XGBoots algorithm includes regularization as part of the learning objective [35].

The last MLT considered belongs to the family of deep learning models, namely the single hidden layer NNET. It is the simplest form of the NNET, in which there is only one layer of input nodes that send weighted inputs to a subsequent layer of receiving nodes [32].

### 2.6. Model Optimization and Predicted Probability Threshold

The models were developed using 70% of the total sample (training set) and tested on the remaining 30% (test set). Subjects for the training and test sets were selected randomly while maintaining the proportion of events (NIV failures), with a seed set to ensure reproducibility.

Each model was optimized on the training set using 10-fold cross-validation. Specifically, the model was trained on 9 folds, with the remaining fold used as the validation set. This process was repeated 10 times, ensuring that each fold was used as the validation set once. The results of the models were averaged across all 10 iterations to provide a robust estimate.

The final model performances was then assessed on the test set. A threshold determined by Youden’s J statistic [36]—corresponding to the maximum sum of specificity and sensitivity—was used to assign each subject to the predicted class (NIV success or failure). Consequently, the confusion matrix of predicted versus observed probabilities was constructed for model evaluation.

### 2.7. Statistical Analysis

Descriptive statistics were reported as I-quartile/median/III-quartile for continuous variables and as percentages (absolute numbers) for categorical variables. In addition, Wilcoxon-type tests were performed for continuous variables and Pearson Chi-squared test or Fisher exact test, as appropriate, for categorical variables. The level of significance was set at 0.05.

The classification ability of the models was estimated by the sensitivity, specificity, negative (NPV), and positive (PPV) predictive values. The discrimination ability was estimated by the area under the receiver operating characteristics (AUROC), while the calibration was checked through calibration plot, the average (Eavg), the median (E50), and the 90th percentile(E90) of the absolute difference between predicted and observed probabilities. The performance measures were estimated together with the 95% confidence interval.

The VIMP measure, based on the Gini Impurity Index [37], was reported. VIMP quantifies the contribution of each predictor in predicting the outcome, providing a ranking of the most important variables [38].

The analyses were performed using R Statistical Software 4.3.0 (Vienna, Austria) [39] with the SuperLearner [33], randomForest [40], and xgboost [41], nnet [42], caret [43], pmcalibration [44], and betacal [45] packages.

## 3. Results

From 43,794 records, 1861 patients met the inclusion criteria, of whom 241 experienced NIV failure (13%) (Figure S1).

The most common NIV failure reason was hypoxia with 177 cases, followed by hypercapnia and excessive secretions with 122 and 42 cases, respectively (Table S1). Moreover, 233 (96.7%) patients with NIV failure at the first attempt required a subsequent intubation, and 31 (13%) died in the PICU, compared to seven children (0.4%) in the NIV success group.

Table 1 shows the demographic data, clinical history and characteristics at presentation of the sample according to outcome development.

Table 2 reports the different devices used in the study population for NIV delivery. Oronasal masks, nasal cannulas and full-face (eyes included) devices appear more frequently in the NIV failure group compared to the NIV success group. On the other hand, nasal masks and helmets are similarly distributed among the groups.

Table 3 reports the NIV failure timing: 85 (35%) failures happened within the first 24 hours (h), 174 (72%) within 48 h, and 203 (84%) within 3 days after initiating NIV.

FiO_2_, priority of admission and the POPC, present the highest amount of missing values, 56.3%, 21.3%, and 9.8%, respectively. After listwise deletion of missing values, the original sample decreased to 678 observations with 97 NIV failure cases (14.3%). There was no statistical difference in NIV failure population characteristics between overall cases and complete cases only (Table S2). Table 4 shows the results of MLTs on the complete case dataset.

**Figure 2:**
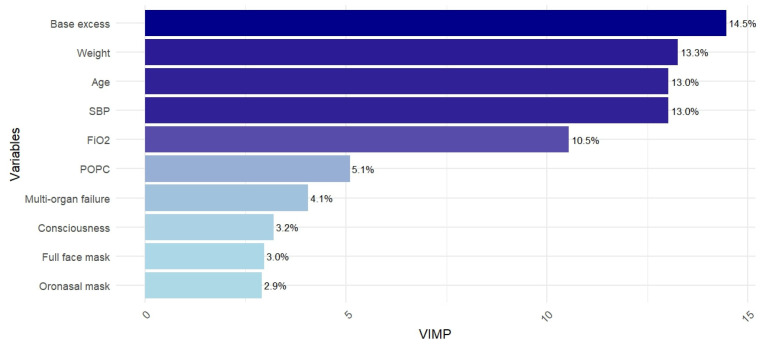
VIMP (%) calculated by the RF model for the top 10 ranked predictors. Each bar is colored according to its importance: dark blue (higher importance), light blue (lower importance).

Figure 2 shows the VIMP (in percentage on the cumulative variable importance estimations) for the top 10 predictors in the RF model.

**Figure 3:**
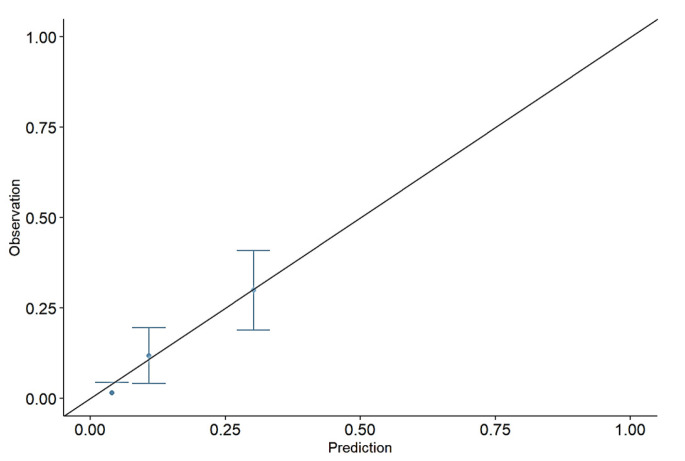
Calibration plot using predicted probabilities by RF model.

Finally, the calibration metrics were estimated by the RF model equal to Eavg = 0.2, E50 = 0.11, and E90 = 0.61. The corresponding calibration plot is reported in Figure 3.

## 4. Discussion

### 4.1. Main Results

This study aimed to compare the performances of diverse MLTs in predicting first-attempt NIV failure during PICU stay in patients enrolled in the TIPNet registry, particularly in the context of low event rates. The models were evaluated based on their ability to accurately classify rare positive cases, with a priority given to higher sensitivity over AUROC. Among the various models tested, the RF model exhibited the highest sensitivity, recorded at 0.83 (95% CI: 0.64, 0.94), with a corresponding AUROC of 0.82 (95% CI: 0.74, 0.90). This sensitivity surpassed that reported by Bose et al. [25] and matched the sensitivity reported by Feng et al. [23], though it was achieved with a less complex model. Although the bagging algorithm may be considered less sophisticated than the boosting algorithm, RF outperformed XGBoost in this analysis. One possible explanation for this result could be the presence of a relatively low number of predictors (19); therefore, the regularization inherent in XGBoost could negatively rather than positively affect its ability to generalize when applied to data without overfitting problems (underfitting). A similar note can be made for the NNET model: the more complicated and sophisticated algorithm tends to generalize worse (overfitting) when applied to a relatively simple dataset.

From a clinical standpoint, the predictors were entered into the model, and all their additive and interaction effects were considered, thanks to the application of MLTs rather than the simple additive logistic regression model [46]. The VIMP highlights that base excess in the first hour, age at admission, weight at admission, SBP in the first hour, and FiO_2_ in the first hour are the predictors that contributed more to the NIV failure prediction. Interestingly, base excess, weight, age, and SBP showed no statistically significant differences in distributions between the failure and success groups, as shown in Table 1. However, they emerged as the most important variables in the VIMP analysis when included in the RF model. This underscores the importance of accounting for variable interactions and non-linear relationships in predicting outcomes for complex patients, such as children in the PICU setting. Nevertheless, base excess, SBP, and FiO_2_ stand out as both reasonable and literature-supported predictors of NIV failure [21,47,48]. In fact, base excess is an indicator of metabolic status, with deviations often reflecting systemic acidosis and poor respiratory compensation [49]. Similarly, unstable SBP suggests a hemodynamic compromise [8,50], while elevated FiO_2_ requirements point to significant hypoxemia and respiratory strain [8].

### 4.2. The Model Calibration

Although a classification model might seem more practical than a scoring model, it can be considered a “premature” choice because it overrides the decision maker’s role in determining the costs associated with incorrect decisions [51]. To achieve optimal decisions, it is essential to fully utilize available data, develop accurate predictions, and apply utility/cost functions to make final decisions (classification). Since end users have diverse utility functions, providing a classification model erroneously presumes that all users share the same utility function and that the one inferred by the classification system is universally applicable. Thus, the event probability estimator should always be provided to end users, such as PICU physicians. In light of this, along with the classification model, we have included the calibration assessment to ensure alignment between predicted and observed probabilities within the sample. Moreover, we suggest always checking for calibrated probabilities when making predictions on diverse target populations.

### 4.3. Study Limitation

The main limitation of this study is the absence of variables that report specific disease indicators such as respiratory rate, heart rate, presence of tachypnoea, and oxygen saturation from pulse oximetry [8]. This lack of detailed clinical data could affect the model’s ability to capture all relevant aspects of the patient’s condition. However, the good performance of the model achieved even when using only general predictors could provide a promising tool for risk stratification even in clinical settings with few resources and few technological advances [52]. Additionally, the fixed nature of the current risk score could be enhanced by incorporating time-dependent covariates, allowing for a dynamic assessment of the risk of events over time. This approach could provide a more nuanced and accurate prediction model, particularly for failures that occur after 24 h of initiating NIV [23].

### 4.4. Final Remarks

The study successfully identified the most important predictors of NIV failure in TIPNET patients, with RF emerging as a particularly sensitive model. Moreover, the model calibration ensures that the predicted probabilities are reliable and clinically useful for stratifying the risk of NIV failure in the target population. However, future research should focus on incorporating more specific disease indicators and dynamic risk assessment methods to enhance model utility, improving the prevention of rare events in the PICU setting both at admission and during the follow-up.

## Figures and Tables

**Table 1:** Subjects’ baseline characteristics according to outcome development.

Characteristics	NIV Failure, *N* = 241 ^1^	NIV Success, *N* = 1620 ^1^	*p*-Value ^2^	q-Value ^3^
Age (months)	32.94 (2.50, 39.90)	25.77 (2.40, 26.90)	0.071	0.10
Sex (Female)	123 (51%)	897 (55%)	0.2	0.3
Weight	12.26 (4.70, 13.00)	10.93 (5.00, 12.00)	0.6	0.7
Ethnicity			0.038	0.059
⠀⠀Caucasian	194 (80%)	1204 (74%)		
⠀⠀Other	47 (20%)	416 (26%)		
Chronic disease	105 (44%)	468 (29%)	<0.001	<0.001
Systolic Blood Pressure (SBP)	102.56 (90.00, 119.00)	100.78 (90.00, 117.00)	0.3	0.3
⠀⠀(Missing)	4	22		
FiO_2_ (fraction of inspired oxygen)	0.54 (0.40, 0.60)	0.58 (0.30, 0.50)	<0.001	<0.001
⠀⠀(Missing)	120	928		
Base excess	2.28 (−2.00, 2.30)	0.31 (−0.85, 1.60)	>0.9	>0.9
⠀⠀(Missing)	4	44		
Priority of admission			0.001	0.002
⠀⠀High	147 (79%)	883 (69%)		
⠀⠀Medium	32 (17%)	365 (29%)		
⠀⠀Low	8 (4.3%)	29 (2.3%)		
⠀⠀(Missing)	54	343		
State of consciousness			<0.001	<0.001
⠀⠀Conscious	177 (73%)	1447 (89%)		
⠀⠀Pharmacological sedation	13 (5.4%)	54 (3.3%)		
⠀⠀Other	51 (21%)	118 (7.3%)		
⠀⠀(Missing)	0	1		
Paediatric overall performance category (POPC—min = 1; max = 6)	2.02 (1.00, 3.00)	1.72 (1.00, 2.00)	0.001	0.002
⠀⠀(Missing)	23	159		
Multiorgan failure	38 (16%)	43 (2.7%)	<0.001	<0.001
Bronchiolitis	89 (37%)	801 (49%)	<0.001	<0.001
Asthma	14 (5.8%)	141 (8.7%)	0.13	0.2

1. Median (IQR); n (%). 2. Wilcoxon rank sum test; Pearson’s Chi-squared test; Fisher’s exact test. 3. False discovery rate correction for multiple testing.

**Table 2:** Type of NIV devices.

Characteristic	NIV Failure, *N* = 241 ^1^	NIV Success, *N* = 1620 ^1^	*p*-Value ^2^	q-Value ^3^
Nasal mask	36 (15%)	287 (18%)	0.3	0.3
Oronasal mask	47 (20%)	101 (6.2%)	<0.001	<0.001
Nasal cannulas	60 (25%)	579 (36%)	<0.001	0.002
Helmet	90 (37%)	693 (43%)	0.11	0.15
Full-face (eyes included)	42 (17%)	75 (4.6%)	<0.001	<0.001

1. Median (IQR); n (%). 2. Wilcoxon rank sum test; Pearson’s Chi-squared test; Fisher’s exact test. 3 False discovery rate correction for multiple testing.

**Table 3:** Days before the NIV failure (first attempt).

Time Before Failure	*N* (%)	Cumulative *N* (%)
within 24 h	85 (35%)	85 (35%)
within 48 h	89 (37%)	174 (72%)
within 3 days	29 (12%)	203 (84%)
within 4 days	9 (3.7%)	212 (87.7%)
within 5 days	7 (2.9%)	219 (90.6%)
within 6 days	3 (1.2%)	222 (91.8%)
within 7 days	4 (1.7%)	226 (93.5%)
>7 days	15 (6.5%)	241 (100%)

**Table 4:** Comparison of the performance of the MLTs.

Model	Sensitivity	Specificity	AUROC	PPV/NPV
GLM	0.76 (0.56, 0.90)	0.83 (0.76, 0.88)	0.81 (0.72, 0.91)	0.42/0.95
RF	0.83 (0.64, 0.94)	0.72 (0.65, 0.78)	0.82 (0.74, 0.90)	0.33/0.96
XGBoost	0.72 (0.53, 0.87)	0.70 (0.63, 0.77)	0.72 (0.61, 0.82)	0.29/0.94
NNET	0.62 (0.42, 0.79)	0.52 (0.44, 0.59)	0.50 (0.50, 0.50)	0.18/0.89
SuperLearner	0.76 (0.56, 0.90)	0.82 (0.76, 0.88)	0.82 (0.73,0.92)	0.42/0.95

## Data Availability

The data presented in this study are available upon request from the corresponding author.

## Supplementary Materials

The following supporting information can be downloaded at: https://www.mdpi.com/article/10.3390/diagnostics14242857/s1, Table S1: Reasons of NIV failures (more than one reason per subject); Table S2: Comparison of NIV failure population characteristics between overall cases and complete cases only; Figure S1: Flowchart for patients’ selection.
