# Supplementary material for: Non-Invasive Ventilation Failure in Pediatric ICU: A Machine Learning Driven Prediction
Source: Diagnostics (Basel). 2024 Dec 19;14(24):2857. doi: 10.3390/diagnostics14242857 (PMC11675706; doi:10.3390/diagnostics14242857)
Supplement: Supplementary file 1 [file diagnostics-14-02857-s001.zip › diagnostics-3361815-supplementary.pdf]

diagnostics

MDPI

Supplementary Materials

Non-Invasive Ventilation Failure in Pediatric ICU: A Machine Learning Driven Prediction

Table S1. Reasons of NIV failures (more than one reason per subject).

Characteristic	NIV Failure, N = 241[1]
Hypoxia	177 (73.4%)
Hypercapnia	122 (50.6%)
Excessive secretions	42 (17.4%)
Discomfort/agitation	28 (11.6%)
Ineffective coughing	19 (7.9%)
Intolerance	19 (7.9%)

[1]n (%)

Table S2. Comparison of NIV failure population characteristics between overall cases and complete cases only.

Characteristic	All Cases N = 241[1]	Complete Cases N = 97[1]	p-Value[2]	q-Value[3]
Age (months)	32.94 (2.50, 39.90)	28.33 (2.40, 37.10)	0.7	>0.9
Sex (Female)	123 (51%)	46 (47%)	0.5	>0.9
Weight	12.26 (4.70, 13.00)	11.22 (4.70, 12.00)	0.5	>0.9
Ethnicity			0.7	>0.9
Caucasian	194 (80%)	76 (78%)		
Other	47 (20%)	21 (22%)		
Chronic disease	105 (44%)	40 (41%)	0.7	>0.9
Systolic Blood Pressure (SBP)	102.56 (90.00, 119.00)	101.60 (90.00, 114.00)	0.6	>0.9
(Missing)	4	0		
FiO2 (fraction of inspired oxygen)	0.54 (0.40, 0.60)	0.53 (0.40, 0.60)	0.6	>0.9
(Missing)	120	0		
Base excess	2.28 (-2.00, 2.30)	3.97 (-3.70, 2.80)	0.3	>0.9
(Missing)	4	0		
Priority at admission			0.2	>0.9
High	147 (79%)	83 (86%)		
Medium	32 (17%)	13 (13%)		
Low	8 (4.3%)	1 (1.0%)		
(Missing)	54	0		
State of consciousness			0.5	>0.9
Conscious	177 (73%)	66 (68%)		
Pharmacological sedation	13 (5.4%)	8 (8.2%)		
Other	51 (21%)	23 (24%)		
Paediatric overall performance category (POPC - min=1; max=6)	2.02 (1.00, 3.00)	2.00 (1.00, 3.00)	0.9	>0.9
(Missing)	23	0		
Multi-organ failure	38 (16%)	16 (16%)	0.9	>0.9
Bronchiolitis	89 (37%)	41 (42%)	0.4	>0.9
Asthma	14 (5.8%)	8 (8.2%)	0.4	>0.9

[1] Median (IQR); n (%). [2] Wilcoxon rank sum test; Pearson's Chi-squared test; Fisher's exact test. [3] False discovery rate correction for multiple testing.

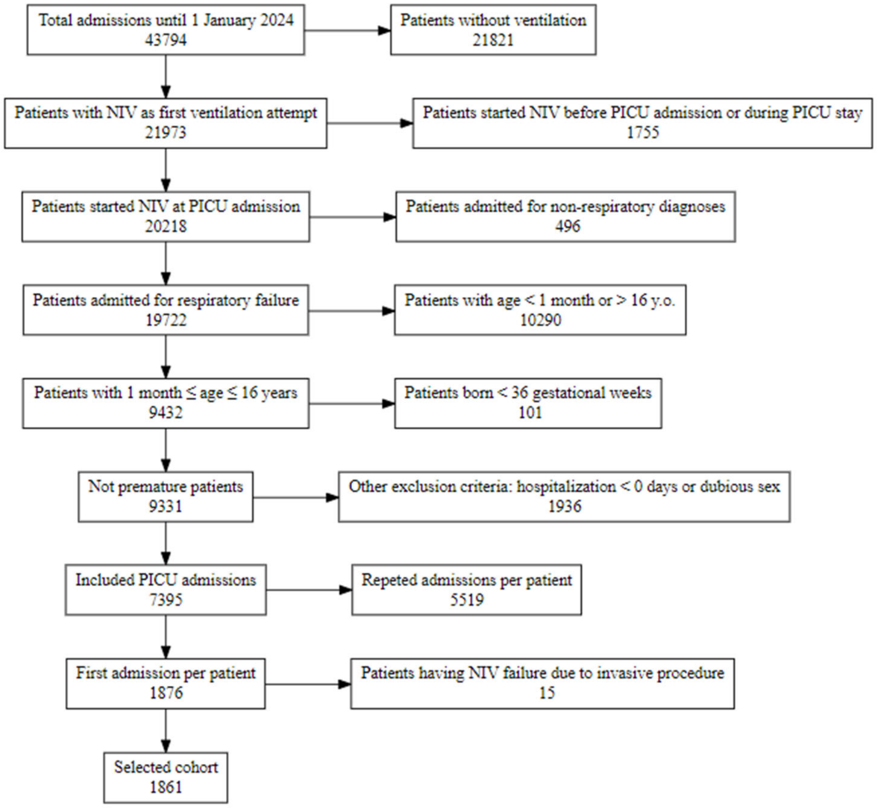

Figure S1. Flowchart for patients' selection.
